# Behavioral and Endocrine Consequences of Simultaneous Exposure to Two Different Stressors in Rats: Interaction or Independence?

**DOI:** 10.1371/journal.pone.0021426

**Published:** 2011-06-24

**Authors:** Cristina Muñoz-Abellán, Cristina Rabasa, Nuria Daviu, Roser Nadal, Antonio Armario

**Affiliations:** 1 Institut de Neurociències, Universitat Autònoma de Barcelona, Bellaterra, Barcelona, Spain; 2 Unitat de Fisiologia Animal, Facultat de Biociències, Universitat Autònoma de Barcelona, Bellaterra, Barcelona, Spain; 3 Unitat de Psicobiologia, Facultat de Psicologia, Universitat Autònoma de Barcelona, Bellaterra, Barcelona, Spain; University of Chicago, United States of America

## Abstract

Although behavioral and endocrine consequences of acute exposure to stressors have been extensively studied, little is known about how simultaneous exposure to two different stressors interacts to induce short- and long-term effects. In the present experiment we studied this interaction in adult male rats exposed to cat fur odor (impregnated cloth) or immobilization on boards either separately or simultaneously. We reasoned that exposure to the odor of a potential predator while immobilized, may potentiate its negative consequences as compared to exposure to only one of the stressors. Exposure to cat odor elicited the expected reduction of activity and avoidance of the area where the impregnated cloth was located. The endocrine response (plasma levels of ACTH and corticosterone, as a measure of the hypothalamic-pituitary-adrenal axis, HPA) was markedly greater after immobilization than after cat fur odor and no additive effects were found by simultaneous exposure to both stressors. Cat odor, but not immobilization, increased anxiety-like behavior as evaluated in the elevated plus-maze 7 days after the stressors, with no evidence of enhanced HPA activation. In addition, cat odor exposure resulted in long-lasting (8 days later) fear conditioning to the box containing a clean cloth, which was reflected by hypoactivity, avoidance of the cloth area and enhanced HPA activation. All these effects were similarly observed in rats exposed simultaneously to cat odor and immobilization. In rats only exposed to immobilization, only some weak behavioral signs of fear conditioning were found, but HPA activation in response to the context paired to immobilization was enhanced to the same extent as in cat odor-exposed animals, supporting a certain degree of endocrine conditioning. The present results did not reveal important behavioral interactions between the two stressors when animals experienced both simultaneously, whereas some interactions were found regarding HPA activation. Theoretical implications are discussed.

## Introduction

Acute exposure to stressors elicits a wide range of physiological and behavioral responses that are markedly dependent on the particular characteristics (i.e. type, intensity and duration) of stressors. The activation of the hypothalamic-pituitary-adrenal (HPA) axis and the subsequent release of ACTH and glucocorticoids (corticosterone in rodents) are the most extensively studied physiological responses to stressors. In addition, it has been repeatedly reported that plasma levels of ACTH and, to a lesser extent, those of corticosterone are good markers of stressor intensity [1].

In nature, organisms are often simultaneously exposed to different types of aversive stimuli (i.e. escape from one or more predators, entering in an unknown environment and risk of pain/injury while rapidly moving in a complex and irregular ground). Under laboratory conditions, simultaneous exposure to different stressors can give us important clues about brain processing of multiple stressors. Quite surprisingly, there is little experimental research on how simultaneous exposure to two stressors interacts to induce short-term and long-term physiological and behavioral effects, including activation of the HPA axis. This interaction is theoretically relevant for several reasons. First, it is unclear whether simultaneous exposure to two stressors may result in predominant processing of the most aversive one, thus reducing the overall impact of the less relevant, or, on the contrary, the impact of the two stressors are added or even potentiated, indicating additive or synergistic effects. Second, if one or the two stressors was capable of developing fear conditioning, would be such conditioning potentiated by simultaneous exposure or some interference would appear due to the development of predominant attention to one of them? To our knowledge, there are no precedents in the field of stress, but studies of fear conditioning indicate that when two conditioned stimuli (CSs) are simultaneously presented there is some competition between CSs to establish association to the unconditioned stimulus (US). Under these conditions, conditioning is stronger to the most salient (i.e. more intense) stimuli and, therefore, conditioning to the other less salient would be lower than if the latter is given alone. This phenomenon has been called overshadowing [2]. Although competition between CSs appears to predominate, under certain conditions potentiation between cues have also been described. If overshadowing or potentiation applies when animals are experiencing two stressors simultaneously, reduced or potentiated conditioning to the less threatening stressor may be predicted.

Two particularly interesting stressors to study this issue are immobilization on boards (IMO) and cat fur odors. Considering all well-accepted physiological markers of stressor intensity, including the activation of the HPA axis, IMO is a severe stressor [3], [4] that can induce in rats long-lasting (days) behavioral and endocrine sensitization [5]–[7] and impairment of spatial memory in the Morris water maze [8]. In mice, prior exposure to IMO can impair extinction of footshock-induced tone fear conditioning [9]. However, we have been unable to establish IMO-induced context fear conditioning using different paradigms (unpublished data). On the contrary, cat fur odor has been found to induce a much lower activation of the HPA axis [7] but strong avoidance, context/cue fear conditioning and long-lasting changes in anxiety-like behavior [10]–[19]. However, the changes in anxiety are less consistent than the former ones and apparently depending on the particular cat used as the source of odor [20]. The presence of cat fur odor signals the proximity of a cat and therefore a life threatening stressor. We reasoned that the detection of this odor when the animals are immobilized, and therefore with no opportunity to escape, may markedly enhance the stressfulness of the situation. We then expected that simultaneous exposure to both stressors may potentiate their acute HPA activation and enhance their long-term consequences (i.e. increase in anxiety as evaluated by a plus-maze, increase in the sensitization of the HPA response to a novel environment, and/or development of behavioural or endocrine fear conditioning). Alternatively, if we assume, on the basis of the physiological response it elicits, that IMO is the most severe stressor, the processing of it may predominate over that of cat odor and negatively interfere with the capability of the latter to induce the above mentioned effects. The behavioural results did not confirm the main hypotheses and indicate that animals are likely to process the two stressors in a quite independent way, whereas some interaction appeared specifically at the level of the HPA axis, particularly during fear conditioning testing.

## Materials and Methods

### Ethics Statement

The experimental protocol was approved by the Committee of Ethics of the Universitat Autònoma de Barcelona (approval ID: CEEAH 3385 and DMAH 4527), followed the “Principles of laboratory animal care” and was carried out in accordance with the European Communities Council Directive (86/609/EEC).

### Animals

We used 40 male Sprague–Dawley rats, 42 days old on the day of cat odor exposure (body weight: 199±3.7 g), obtained from the breeding centre of the Universitat Autònoma de Barcelona. After arrival at the laboratory, they were housed in pairs, in standard conditions of temperature (21±1°C) on a 12-h light/12-h dark schedule (lights on at 0700 h). Food and water were available *ad libitum*.

### General procedure

The experimental procedures were always done in the morning. Starting four days after their arrival, all animals were handled for four days for approximately 2 min a day. After that, blood samples were taken under basal conditions by tail-nick to habituate animals to the procedure. The tail-nick consisted of gently wrapping the animals with a cloth, making a 2 mm incision at the end of one of the tail veins and then massaging the tail while collecting, within 2 min, 300 µl of blood into ice-cold EDTA capillary tubes (Sarsted, Granollers, Spain). This procedure is extensively used in our lab and by others because resting levels of hormones are obtained [6], [21], [22]. The two rats from a cage were sampled simultaneously (two experimenters were sampling at the same time and a third was gently holding the two rats). Animals were assigned at random to the different experimental groups and individually introduced into an open field (OF) for 20 min under four different conditions: (a) with cat bedding and a clean cloth (controls, n = 10); (b) with cat bedding and a cloth impregnated with cat fur/skin odor obtained by rubbing a cat with the cloth for 5 min (cat fur odor, ODOR, n = 10); (c) with cat bedding and a clean cloth while immobilized (IMO, n = 10); and (d) with cat bedding and a cloth impregnated with cat fur/skin odor while immobilized (ODOR+IMO, n = 10). IMO were left to explore freely the OF during the first 5 min block before being immobilized and maintained into the OF for 15 additional min with the nose close to the location of the cloth. Blood samples were taken immediately after the 20 min exposure to the OF and again at 45 min post-stress.

Seven days after stress exposure (during which animals were handled three more times), all groups were tested for 15 min in an elevated plus-maze (EPM) and blood samples were immediately taken. Starting one day after exposure to the EPM, the animals were exposed for 2 consecutive days to 15 min sessions in the OF, but in this case cat bedding and a clean cloth (no odor) was used in all groups. This was done to study fear conditioning induced by the odor and/or immobilization and the extinction process. Exposure to the EPM and the OF lasted 15 min because we have previously observed that HPA activation reflects footshock-induced contextual fear conditioning when exposure lasted for 15 min [23], but not with 5 min exposure (unpublished data). Moreover, maximum corticosterone secretion in response to a brief ACTH release needs around 15 min to reflect ACTH release [24]. After each 15 min OF or EPM exposure, blood samples were immediately taken. To test the animals in both the EPM and the OF test we changed the experimenter who took the animals from the animal room, the test room and the way of transporting the rats for testing, in order to eliminate possible conditioned cue in the case of the EPM and any conditioned cue other than the context itself in the case of the OF.

### Immobilization on wooden boards

Animals were immobilized by taping their four limbs to metal mounts attached to a board. Head movements were restricted with two plastic pieces (7×6 cm) placed on each side of the head and the body was subjected to the board by means of a piece of plastic cloth (10 cm wide) attached with *velcro* that surrounded all the trunk.

### Cat odor exposure and open-field

Two non-castrated 5 years old male cats living in another animal facility (Isoquimen, S.L., Sant Feliu de Codines, Spain) were used as the source of odor and no differences were found between the cloths from each cat in the variables under investigation. Odor clothes were obtained approximately 24 h before the beginning of the experiment and each cloth was separately sealed in air-tight plastic containers maintained at room temperature.

The OF was a plastic gray rectangular box opened at the top (56×36.5×31 cm) with dim illumination provided by a white 25 w bulb placed 1.20 m above the center of the surface of the box. A piece of cotton towel impregnated with cat fur odor or a clean piece (30×23.5 cm) was placed inside a small container (23×13×2 cm) fixed at one end of the box floor. The OF area was divided into three equal zones, being zone 1 (Z1) the one in which the cloth was located, zone 3 (Z3) the opposite to the cloth and zone 2 (Z2) the intermediate one. The two cage-mates always received the same treatment and were simultaneously tested in two separate OF placed in the same room. For behavioral analysis, one video camera (Sony SSC-M388 CE, BW) was suspended from the ceiling (1.20 m above the surface of the OF) that recorded simultaneously the two OF. A digital video recorder (JVC VR-716) sampled the position of the rat (8.3 samples/s) and it was used to transfer the videos to a computer for subsequent video tracking using the center of gravity of the animal (Smart version 2.5.20, Panlab, S.L.U, Barcelona, Spain). An experimenter blind to the treatment obtained from the tracks: (i) the distance travelled, as measures of hypoactivity, and (ii) the time spent in each zone, as measures of aversion to the odor area.

The animals were transported in their home-cages from the vivarium to adjacent test rooms. The experiment was performed in two different, well-ventilated, rooms other than those used to take blood samples and to measure EPM behavior (see below). To prevent cross-contamination on the experimental day, rats exposed to cat odor were run in a separate room than the non-exposed. Across one session, the same cloth was used in a given apparatus for all the rats of the same treatment.

### Elevated plus-maze

The apparatus, adapted from Pellow and File [25] consisted of four white wooden arms (formica) at right angles to each other, connected to a central square (10 cm2) to form the shape of a plus sign and elevated 50 cm above the floor. Each arm was 46 cm long and 10 cm wide. Two of the opposite arms had high walls (enclosed arms, 43 cm high), whereas the other two were the open arms that had a 0.7 cm ridge to provide an additional grip. The rat was placed facing a closed arm and the subject was considered to be in a given arm when all paws were inside. A black curtain surrounded the EPM to minimize external influences and a red 15 w bulb was placed 1.20 m above the centre of the apparatus. The two animals of the same home-cage were tested simultaneously in two separate rooms. In order to avoid any signal associated to stress exposure, the experimenter that handled the animals, the way of transporting the animals, the test room and the room's illumination were different from the odor exposure day. Behavior was videotaped as mentioned for subsequent manual analysis. We measured time spent in open and closed arms, number of entries in each type of arm and defecations. The apparatus was cleaned carefully between animals with a water solution containing ethanol (5%, v/v).

### Biochemical analysis

Plasma ACTH and corticosterone levels were determined by double-antibody radioimmunoassays (RIA). In brief, ACTH RIA used 125I-ACTH (PerkinElmer Life Science, Boston, USA) as the tracer, rat synthetic ACTH 1–39 (Sigma, Barcelona, Spain) as the standard and an antibody raised against rat ACTH (rb7) kindly provided by Dr. W.C. Engeland (Department of Surgery, University of Minnesota, Minneapolis, USA). The characteristics of the antibody have been described previously [26] and we followed a non-equilibrium procedure. Corticosterone RIA used 125I-corticosterone-carboximethyloxime-tyrosine-methyl ester (ICN-Biolink 2000, Barcelona, Spain), synthetic corticosterone (Sigma, Barcelona, Spain) as the standard and an antibody raised in rabbits against corticosterone–carboximethyloxime-BSA kindly provided by Dr. G. Makara (Inst. Exp. Med., Budapest, Hungary). The characteristics of the antibody and the basic RIA procedure have been described previously [27]. All samples to be statistically compared were run in the same assay to avoid inter-assay variability. The intra-assay coefficient of variation was less than 6% for ACTH and corticosterone. The sensitivity was 12.5 pg/ml for ACTH and 0.1 µg/dl for corticosterone.

### Statistical analysis

Data were analyzed by the Statistical Program for Social Sciences (SPSS), version 15. When repeated measures were included in the analysis, we used a generalized linear model repeated measures analysis (generalized estimating equations model, GEE) [28]. Behavioral data during cat odor exposure included time blocks as the within-subjects factor and cat odor as the between-subjects factor (factor IMO was not included as these rats were obviously inactive). Physiological responses after exposure to IMO and cat odor included sampling time as the within-subjects factor (two levels: immediately after stress and 45 min post-stress) and IMO and cat odor (ODOR) as the between-subjects factors (each factor with two levels). The conditioned behavioral and endocrine effects of stress were separately analyzed for each session (day 9 and day 10) and included time blocks as within-subjects factor and IMO and ODOR as between-subjects factors (each factor with two levels). Endocrine data during the two sessions that evaluated conditioning, and the endocrine and behavioral data in the EPM were analyzed with a generalized linear model (GENLIN) [29] with two between-subjects factors: IMO and ODOR. If a statistical significant interaction was found, additional pair-wise comparisons were made. The generalized linear model is a more flexible statistical tool than the standard general lineal model (GLM) because several types of distribution and different covariance structures of the repeated measures data could be chosen. In addition, the generalized linear models do not require homogeneity of variances and admit missing values without removing all data subject. As a method of estimation, the maximum likelihood (ML) was used in all cases. Normality distribution and identity as a link function was always used. The unstructured working correlation matrix for repeated measures was chosen.

## Results

### Acute behavioral response to cat odor

**Figure 1:**
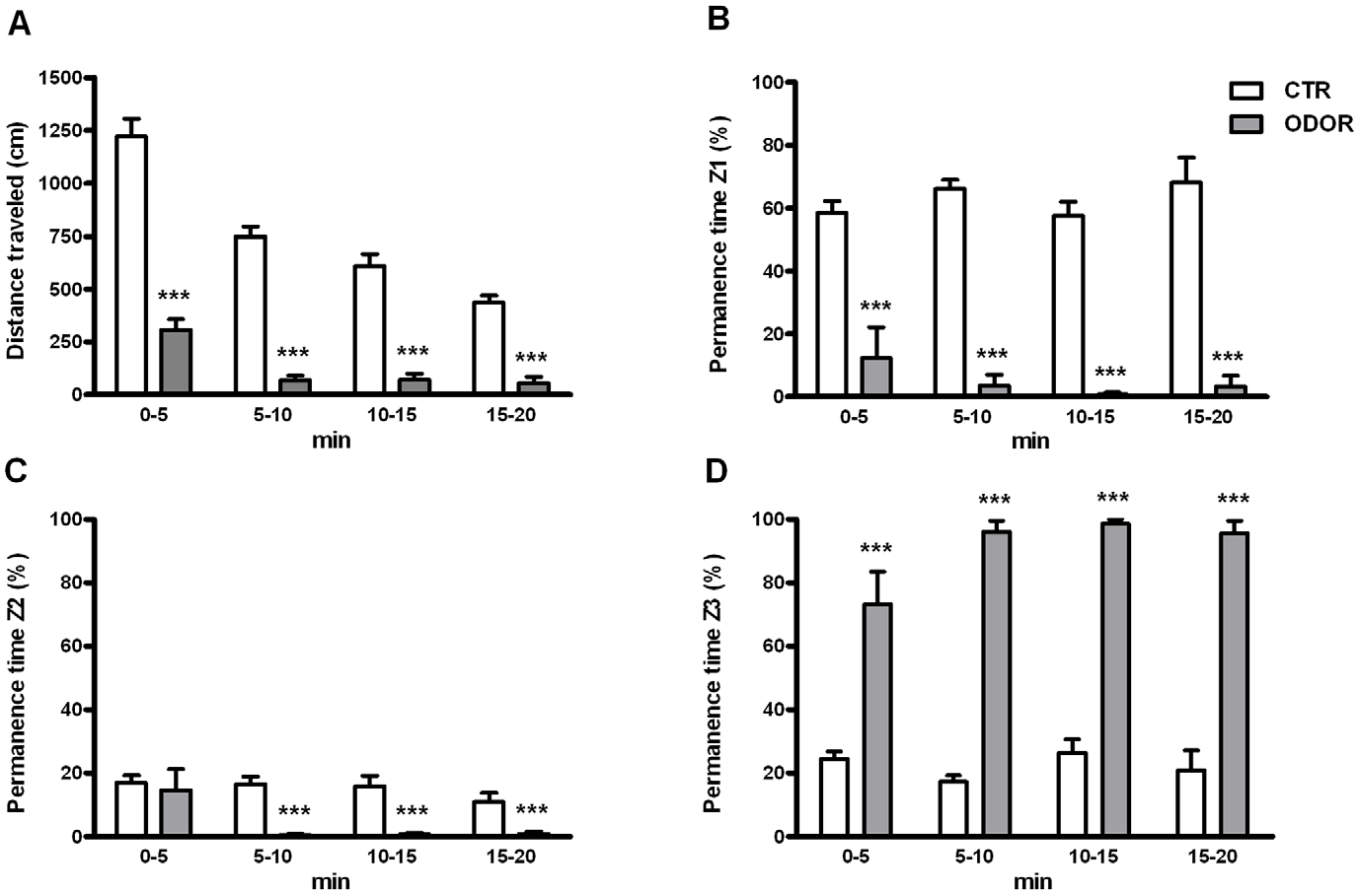
Acute behavioral response to cat odor. Behavioral measures recorded on day 1 during the 20 min exposure to an open field containing a clean cloth (CTR) or a cloth impregnated with cat fur odor (ODOR), expressed by time blocks (5 min each). It's shown the (A) Distance traveled (cm) in the whole of the open field, (B) Permanence time (%) in the zone where the cloth was placed (Z1), (C) Permanence time in the intermediate zone (Z2), and (D) Permanence time in the opposite zone to the cloth (Z3). *** p<0.001 versus non-odor exposed animals.

The statistical analysis of the effects of cat odor on the distance travelled in the OF (Figure 1A) showed significant effects of ODOR (Wald χ2 (1) = 297.57, p<0.001), BLOCK (Wald χ2 (3) = 105.16, p<0.001) and ODOR×BLOCK (Wald χ2 (3) = 36.64, p<0.001). Decomposition of the interaction ODOR×BLOCK indicated that hypoactivity was maintained across the 4 blocks of 5 min (p<0.001 for all time blocks), but group differences progressively diminished. The analysis of percent time spent around the place where the odor impregnated cloth was located (Z1) revealed strong avoidance that was slightly increased across time [Figure 1B, ODOR: Wald χ2 (1) = 113.23, p<0.001 and ODOR×BLOCK: Wald χ2 (3) = 14.50, p<0.01]. Consequently, odor exposed rats showed preference for the area (Z3) opposite to the cloth [Figure 1D, ODOR: Wald χ2 (1) = 171.76, p<0.001, BLOCK: Wald χ2 (3) = 8.54, p<0.05 and ODOR×BLOCK: Wald χ2 (3) = 21.36, p<0.001]. The interaction ODOR×BLOCK in Z1 and Z3 reflected that differences between control and cat odor groups increased rather than decreased across time. Similar results were obtained with regard to the percent of time spent in Z2 [Figure 1C, ODOR: Wald χ2 (1) = 18.48, p<0.001, BLOCK: Wald χ2 (3) = 9.06, p<0.05 and ODOR×BLOCK: Wald χ2 (3) = 8.30, p<0.05]. Decomposition of the interaction ODOR×BLOCK revealed that differences were not statistically significant during the first block and they were only observed from the second to the last block (p<0.001 in all cases).

Since IMO and ODOR+IMO groups were exposed to the OF for 5 min (with or without the cat odor) before the immobilization procedure was done, we directly tested if the groups of IMO and non-IMO rats were statistically similar. The analysis indicated that rats to be immobilized showed comparable levels of motor activity and equal time spent in the different zones in comparison to the corresponding non-IMO animals (data not shown).

### Acute endocrine response to cat odor and immobilization

**Figure 2:**
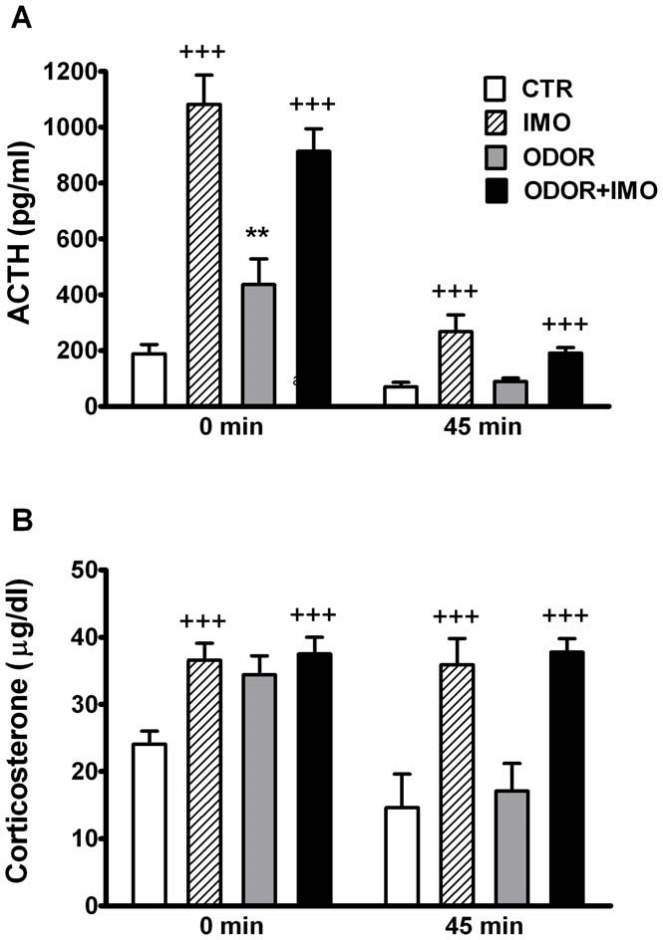
Acute endocrine response to cat odor and immobilization. Plasma (A) ACTH and (B) Corticosterone levels of rats exposed for 20 min to the open-field (CTR), exposed 5 min to the open-field and then immobilized and maintained within the open-field (IMO), exposed for 20 min to the open-field containing cat fur odor (ODOR), or exposed to the open-field containing cat fur odor for 5 min, then immobilized and maintained for 15 min in the same open-field (ODOR+IMO). Samples were taken immediately after stress (0 min) and at 45 min post-stress. ^+++^ p<0.001 versus non-IMO exposed animals; ** p<0.05 versus non-odor exposed animals.

The statistical analysis of ACTH levels in response to cat odor and/or IMO exposure (Figure 2A) showed significant effects of IMO (Wald χ^2^ (1) = 88.44, p<0.001), TIME (Wald χ^2^ (1) = 163.97, p<0.001), IMO×TIME (Wald χ^2^ (1) = 46.88, p<0.001), ODOR × IMO (Wald χ^2^ (1) = 8.35, p<0.01) and ODOR × IMO × TIME (Wald χ^2^ (1) = 4.25, p<0.05), but not of ODOR and ODOR ×TIME. Decomposition of the interaction ODOR × IMO × TIME showed that: (i) IMO induced an increase in ACTH when compared to control animals both immediately after stress exposure (0 min) and at 45 min post-stress (p<0.001 at both times); (ii) cat odor increased ACTH levels with respect to the control group, but the effect was only observed immediately after stress exposure (p<0.01); (iii) ACTH levels in ODOR+IMO animals were similar to those observed in IMO animals and higher than in ODOR animals after stress and at 45 min post-stress (p<0.001 at both times).

When analyzing corticosterone levels (Figure 2B), the statistical analysis revealed significant effects of IMO (Wald χ^2^ (1) = 36.58, p<0.001), TIME (Wald χ^2^ (1) = 17.80, p<0.001) and IMO × TIME (Wald χ^2^ (1) = 18.83, p<0.001), but not of ODOR or other interactions. Decomposition of the interaction IMO × TIME showed that IMO exposure increased corticosterone in comparison to non-exposed IMO animals and this increase was evident after the end of the stress and even more at 45 min post-stress, when corticosterone levels had decline in non-IMO groups but were maintained in IMO groups (p<0.001 in both times). Although the global statistical analysis did not reveal an ODOR effect, a non-significant trend to increase corticosterone levels was found for cat odor exposed groups in comparison to non-exposed odor groups. In fact a direct comparison of the ODOR group with controls immediately after stress exposure revealed a significant difference (p<0.01).

### Re-exposure to the context

**Figure 3:**
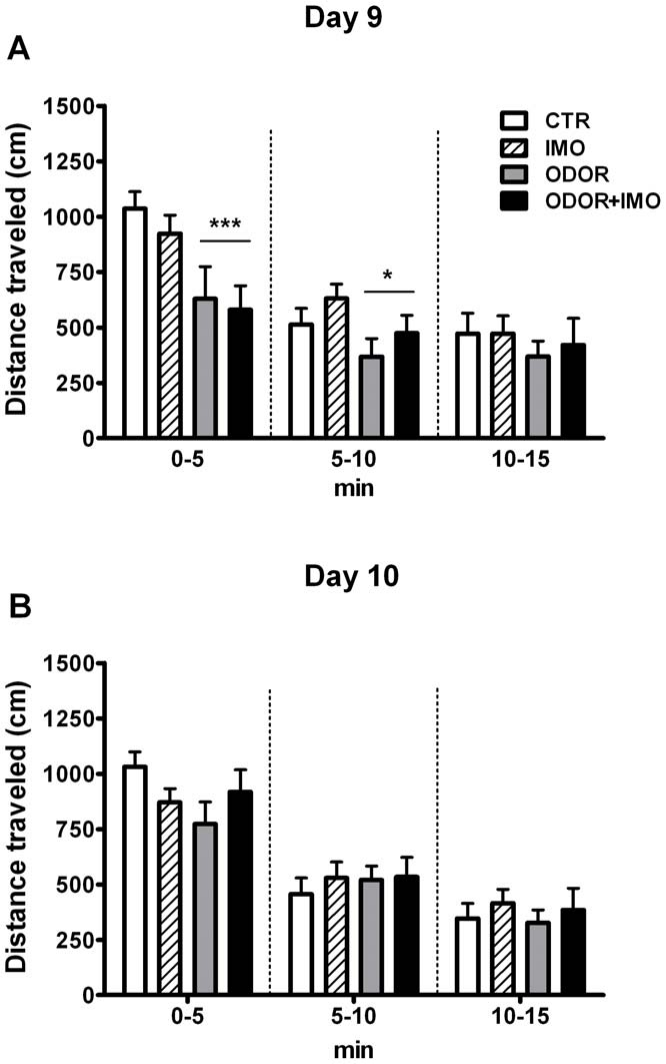
Distance travelled during the re-exposure to the context. Starting 8 days after exposure to stress, control (CTR), cat fur odor (ODOR), immobilization (IMO) and cat fur odor+immobilization (ODOR+IMO) rats were re-exposed to the stress-paired context. Panel A shows the distance traveled during the first context re-exposure on day 9, divided by time blocks (5 min each) and Panel B the same parameter during the second context re-exposure on day 10. * p<0.05; *** p<0.001 versus non-odor exposed animals.

The statistical analysis of the distance travelled in the OF during the first exposure to the context on day 9 (Figure 3A) showed significant effects of ODOR (Wald χ^2^ (1) = 8.10, p<0.01), BLOCK (Wald χ^2^ (2) = 51.14, p<0.001) and ODOR × BLOCK (Wald χ^2^ (2) = 8.25, p<0.05), but not of IMO or other interactions. Decomposition of the interaction ODOR × BLOCK showed that the differences between odor exposed and non-exposed animals were already evident during the first time block (p<0.001), were still maintained in the second block (p<0.05) and disappeared in the last block. When analyzing the distance travelled during the second exposure to the context on day 10 (Figure 3B), only a significant effect of BLOCK was found (Wald χ^2^ (2) = 159.2, p<0.001), all the other factors and interactions being non-significant. Thus, odor-induced hypoactivity was extinguished by the second context session.

**Figure 4:**
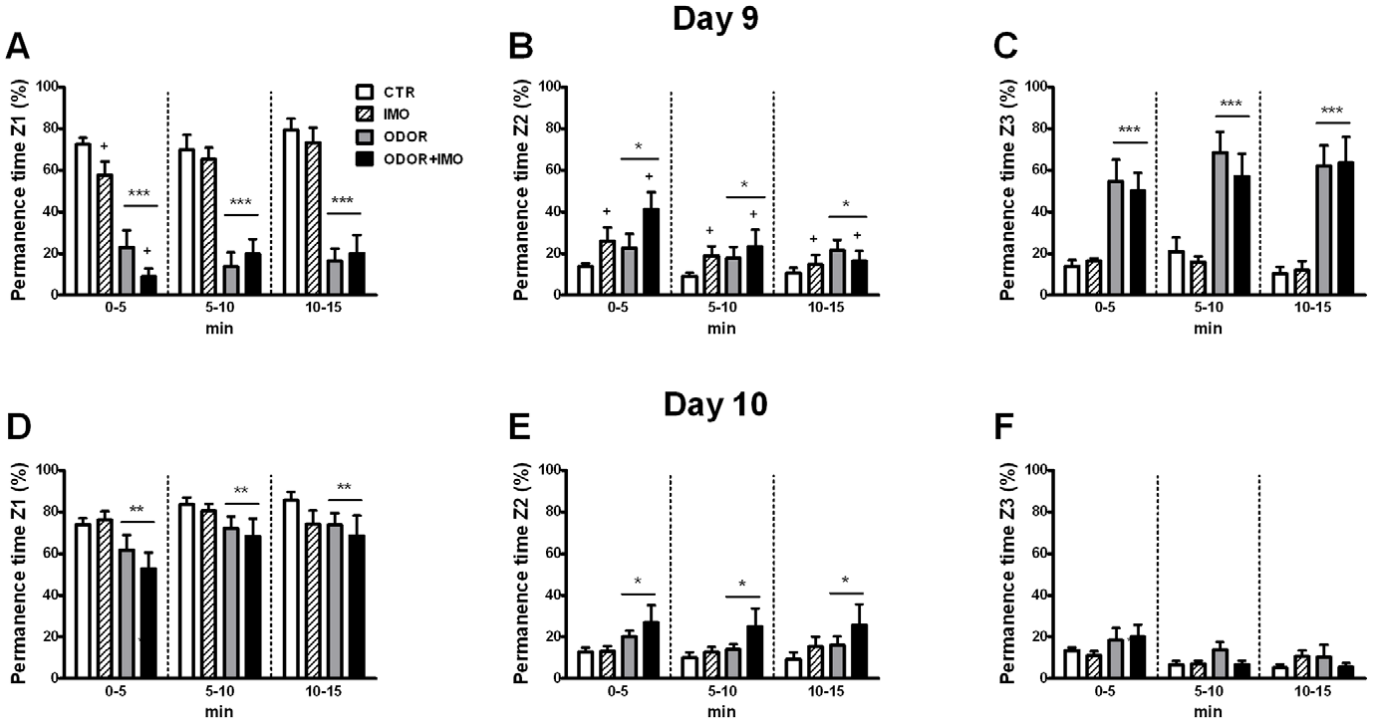
Time spent in the different zones of the open-field during the re-exposure to the context. Starting 8 days after exposure to stress, control (CTR), cat fur odor (ODOR), immobilization (IMO) and cat fur odor+immobilization (ODOR+IMO) rats were re-exposed to the stress-paired context. Then, permanence time (%), divided by time blocks (5 min each), was measured on day 9 in (A) the zone where the cloth was placed (Z1), (B) the intermediate zone (Z2), and (C) the zone opposite to the cloth (Z3). The same parameters were again measured on day 10 (panels D–F). ^+^ p<0.05 versus non-IMO animals; * p<0.05, ** p<0.01, *** p<0.001 versus non-odor exposed animals.

The analysis of the percent time in Z1 during the first context session on day 9 (Figure 4A) revealed significant effects of ODOR (Wald χ^2^ (1) = 104.11, p<0.001) and the interaction IMO×BLOCK (Wald χ^2^ (2) = 10.31, p<0.01), but not of other factors or interactions. The ODOR effect indicated that the decrease in the time spent in the zone where the cloth was located was maintained across the 3 time blocks in odor-exposed as compared to non-exposed animals. Regardless of odor exposure, the decomposition of the interaction IMO×BLOCK showed a significant reduction of time spent in Z1 in IMO as compared to non-IMO rats only during the first time block (p<0.05). Both cat odor and IMO increased the time spent in Z2 [Figure 4B, ODOR: Wald χ^2^ (1) = 4.97, p<0.05, IMO: Wald χ^2^ (1) = 4.02, p<0.05], although in general permanence diminished over time similarly in all groups [BLOCK: Wald χ^2^ (2) = 9.39, p<0.01]. In contrast, only cat odor increased permanence in Z3 [ODOR: Wald χ^2^ (1) = 53.24, p<0.001] along the 3 time blocks (p<0.001 in all cases). No other factor or interaction was statistically significant.

The analysis of the percent of time in Z1 during the second context session on day 10 (Figure 4D) revealed significant effects of ODOR and BLOCK [ODOR: Wald χ^2^ (1) = 6.87, p<0.01, BLOCK: Wald χ^2^ (2) = 18.56, p<0.001], but not of IMO or the interaction between factors. As expected, odor exposure reduced time in Z1. Similarly, only cat odor increased the time spent in Z2 [ODOR: Wald χ^2^ (1) = 4.19, p<0.05] that was maintained along the 3 time blocks (Figure 4E). When analyzing the time spent in Z3 (Figure 4F), increase in the permanence time produced by odor exposure was no longer observed, only a significant BLOCK effect being observed (Wald χ^2^ (2) = 16.99, p<0.001) that reflected reduction across time.

**Figure 5:**
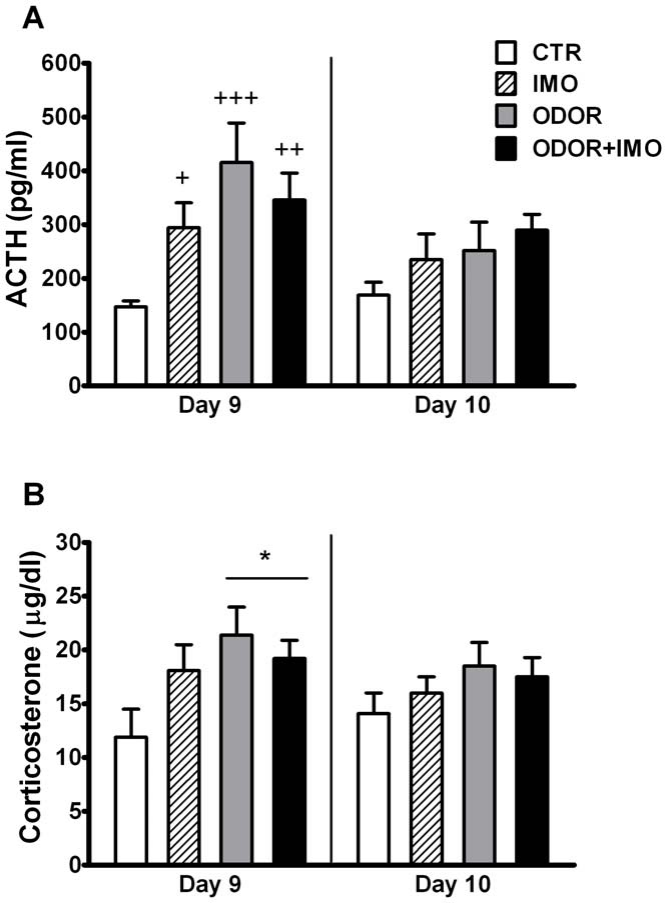
Physiological response to the context re-exposure. Plasma (A) ACTH and (B) Corticosterone responses to the 15 min re-exposure to the context on days 9 and 10, for control (CTR), cat fur odor (ODOR), immobilized (IMO) and cat fur odor+immobilized (ODOR+IMO) rats. ^+^ p<0.05, ^++^ p<0.01, ^+++^ p<0.001 versus CTR (panel A); * p<0.05 versus non-odor exposed groups (panel B).

The statistical analysis of ACTH levels following the first exposure to the context on day 9 (Figure 5A, left) showed significant effects of ODOR (Wald χ^2^ (1) = 10.59, p<0.001) and the interaction ODOR×IMO (Wald χ^2^ (1) = 4.89, p<0.05), but not of IMO. Decomposition of the interaction showed that both ODOR and IMO groups increased the ACTH response to the first context session in comparison to the control group (p<0.001 for ODOR, p<0.05 for IMO), with no additive effect of exposure to ODOR+IMO, which showed significantly higher ACTH levels than controls (p<0.01). However, the statistical analysis of day 9 corticosterone (Figure 5B, left) only indicated a significant increase induced by previous cat odor exposure [ODOR: Wald χ^2^ (1) = 5.81, p<0.05], but not IMO or the interaction ODOR × IMO. The analysis of ACTH and corticosterone responses to the second context session on day 10 revealed no effects of either ODOR or IMO (Figures 5A and 5B, right).

### Long-term effects in the elevated plus-maze

**Figure 6:**
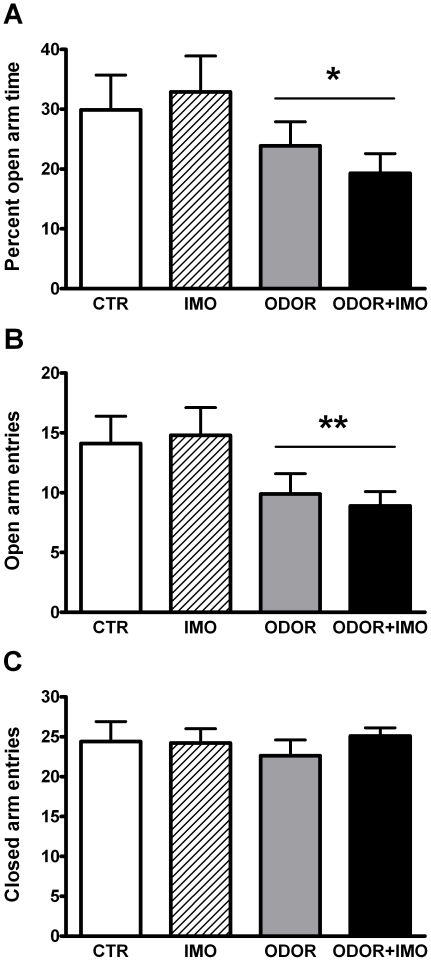
Long-term effects of stress in the elevated plus-maze (EPM). Animals were exposed for 15 min to the EPM 7 days after initial exposure to the different stressors: none (CTR), cat fur odor (ODOR), immobilization (IMO) or cat fur odor+immobilization (ODOR+IMO). (A) Percent of time spent in the open arm, (B) Open arm entries, (C) Closed arm entries. * p<0.05, ** p<0.01 versus non-odor exposed animals.

As shown in Figure 6, one week after exposure to stress, odor-exposed rats showed anxiogenic-like behavior in the EPM. Cat odor exposure resulted in reduced percent of open arm time [Figure 6A, ODOR: Wald χ^2^ (1) = 4.74, p<0.05] and open arm entries [Figure 6B, ODOR: Wald χ^2^ (1) = 8.10, p<0.01]. The other factors and interactions were not significant. The number of closed arms entries (Figure 6C), reflecting general activity, was not affected by the treatments. No differences among the groups were found in ACTH and corticosterone responses to the test (Table 1).

**Table 1:** Endocrine response to the elevated plus-maze (EPM).

	CTR	IMO	CAT	CAT+IMO
	Mean ± SEM	Mean ± SEM	Mean ± SEM	Mean ± SEM
ACTH (pg/ml)	111±16	167±30	134±16	140±15
Corticosterone (µg/dl)	12,8±2,0	15,0±3,5	14,1±2,3	12,9±2,2

Animals were exposed for 15 min to the EPM 7 days after initial exposure to the different stressors: none (CTR), cat fur odor (ODOR), immobilization (IMO) or cat fur odor+immobilization (ODOR+IMO). Plasma ACTH and Corticosterone responses to the EPM were measured. Basal levels taken after the handling period were 20±1 pg/ml for ACTH and 0.9±0.2 µg/dl for corticosterone. No significant differences between groups were found.

## Discussion

The present study was specifically designed to study the unexplored topic of simultaneous exposure to two different stressors and how they interact with each other. We chose two stressors well-characterized in our lab, IMO and cat fur odors, which greatly differ in a wide range of properties. We found that cat fur odor elicited a modest activation of the HPA axis, whereas IMO elicited a much stronger response that did not further increase by simultaneous exposure to both stressors. Cat fur odor, in contrast to IMO, caused strong long-lasting fear conditioning, and enhanced anxiety-like behavior, but such effects were not modified significantly by simultaneous exposure to both stressors. Apparently, in contradiction with our initial hypotheses, there appears to be little interaction between the two stressors regarding their behavioral and physiological consequences.

Whereas much attention has been paid in the field of stress to the topic of how previous exposure to acute and chronic stressors can affect behavioral and physiological response to further superimposed stressors, the consequences of simultaneous exposure to two different stressors are basically unexplored. In the present work, we compared the effects of separate and simultaneous exposure to IMO and cat fur odor. Animals were assigned to four groups and all were exposed for 5 min to a rectangular OF containing either a clean cloth or a cloth previously impregnated with the odor from cat fur. After this initial 5 min period, half of the rats remained as in the previous conditions for 15 additional min whereas the other half were immobilized and returned to the OF for 15 min with the nose close to the location of the cloth. As it has been repeatedly described using footshock that rats cannot develop contextual fear conditioning if shocked immediately after exposure to the chamber (immediate shock effect) [30], we allowed the rats to explore the OF for 5 min before IMO. The two groups of rats exposed to the cat odor showed strong hypoactivity and avoidance of the cloth area during the first 5 min and these effects were maintained over the next 15 min in the group that was not further immobilized, in accordance with previous data [14], [31]–[33].

A significantly greater ACTH response was observed in rats exposed to cat odor as compared to that merely exposed to the OF, but the response to IMO was much greater than that to cat odor. All these data are in accordance with our previous results and those of others after cat or cat odor exposure [7], [16], [20], [34]–[36]. Interestingly, nor synergistic neither additive effect of simultaneous exposure to both stressors was found on plasma ACTH and corticosterone. It may be argued that exposure to IMO already caused a maximum activation, but no differences were either noted during the post-stress period when ACTH levels were clearly submaximal. In addition, a trend toward a lower rather than higher response was observed in animals exposed to both IMO and cat odors. Therefore, it appears that some interference may exist between different stressors regarding brain circuits controlling the activation of the HPA axis. One possibility is that IMO is so severe that its brain processing strongly predominates over that of cat odor and exposure to the latter becomes irrelevant under these circumstances. However this explanation is unlikely considering that fear conditioning to cat odor was not reduced in the rats also exposed to IMO. Then, the possible interference between the stressors may have specifically affected the activation of the HPA axis but not other consequences. At present, there are no studies on this subject, but we can tentatively hypothesize that the population of neurons of the paraventricular nucleus of the hypothalamus (PVN), the critical brain region for the control of the HPA axis, activated by the two stressors overlaps so that no additive effects are found. The extent to which PVN neurons can be activated by any type of stressors or they exhibit some type of specificity linked to the receipt of specific inputs from specific stress circuits is at present unknown. In this regard, it is of interest that the number of corticotropin-releasing hormone (CRH) neurons in the PVN activated by IMO, as revealed by the induction of the immediate early gene c-fos, is only about the fifty percent of the total number of CRH neurons [37]. Since CRH is the main factor controlling the HPA axis and IMO is a severe stressor, it is possible that the population of CRH neurons that were not activated by IMO may respond to other types of stressors, probably systemic stressors.

To study the development of fear conditioning, 8 days after the initial exposure to the stressors, rats were again exposed for 15 min to the same context, including the bedding and a clean cloth without odor. Prior cat odor exposure induced clear signs of fear conditioning. Thus, the two cat odor groups showed hypoactivity with no differences between rats only exposed to cat odor and that exposed to both cat odor and IMO. Such odor-conditioned hypoactivity was only observed during the two first 5 min periods of exposure to the context. In contrast, avoidance of the cloth area was maintained over all periods with no evidence of decline. In addition, IMO-exposed rats showed a small and transient increase in avoidance of the cloth/IMO area, suggesting weak conditioning. Cat odor-induced fear conditioning is in accordance with some previous reports [10], [13], [14], [16], [17], [20], [38] and the present results confirm the reliability of the phenomenon. However, under the very same conditions we were unable to find robust evidence for fear conditioning to IMO. In fact, difficulties to induce IMO-induced contextual fear conditioning have been repeatedly encountered in our lab even in the presence of the IMO board during testing (unpublished data). There is no clear explanation for the lack of conditioning to IMO, but we suspect that cue/contextual fear conditioning is easily established only with some particular types of stressors, as it is the case of footshock or cat odors, in accordance with the concept of biological preparedness that predispose to certain association in function of their biological significance [39]. In particular, contextual fear conditioning appears to be easily established only when the context (i.e. the electrified grids) is an intrinsic component of the stressor.

To rule out that simultaneous exposure to IMO and cat odor could have altered the extinction process, rats were exposed to an additional session in the OF without cat odor. In this second exposure, hypoactivity did not differ among the groups, whereas a certain level of avoidance was still found in the two odor-exposed groups, regardless of exposure to IMO. These data suggest that cat odor-induced fear conditioning is quickly extinguished. Previous work with a very similar procedure has demonstrated a slower rate of extinction that depended on the particular type of behavior measured [10] and was negatively related to the amount of odor-impregnated cloth used [17]. Also a slower extinction was found in adolescent rats using exposure to a cat collar [40]. It is difficult to determine the reason for the differences in the rate of extinction considering the uncertainty regarding the precise odorant molecule involved. However, very recently, some cat kairomone-like ligands, homologs of the major urinary proteins (mup) family, have been detected in the cat saliva [41]. These substances were found to induce, through its detection by the vomeronasal organ (VNO) sensory system, defensive behaviours and ACTH release in mice, similar to those effects observed with cat odors. Therefore, differences in the level of these kairomone chemosignals present in the cat clothes, together with strain and age differences among rats may be involved in those discrepancies in the rate of extinction or other long-lasting behavioural effects.

When long-lasting unconditioned effects of exposure to cat odor and IMO were studied in the EPM, it was found that odor exposure, but not IMO, significantly reduced number of entries and time spent in the open arms, without altering entries in the closed arms. Therefore, cat odor exposure induced a long-lasting increase in anxiety-like behavior, in accordance with previous reports using cat odor exposures [7], [15], [42], [43], although the magnitude of the changes appears to be strongly dependent on the cat used as the source of odor and other unknown factors [20]. Similar long-lasting changes in anxiety have been found after direct exposure of rats or mice to a cat [11], [12], [44]–[49]. It is noteworthy that exposure to IMO, which caused a marked activation of the HPA axis, did not cause a long-lasting enhancement of anxiety and resulted in weak contextual fear conditioning, supporting again the dissociation between the intensity of stressors in physiological terms and their long-lasting behavioral consequences. Marked long-lasting effects of prior exposure to IMO have been reported on sensitization of the HPA and emotional response to superimposed stressors [5], spatial memory [8] and extinction of fear conditioning [9]. Therefore, it is difficult to explain the lack of long-lasting effects of IMO on anxiety as evaluated in the EPM, particularly when compared to other apparently less severe stressors. One can speculate that there is some relationship between this lack of effect on anxiety in novel environments and the difficulties to establish fear conditioning, but this remains to be tested.

The HPA response to the EPM and the context was also evaluated to know whether or not HPA hormones can reflect changes in behavior. No significant differences between groups were found in the EPM, indicating that differences in anxiety were not reflected in a differential activation of the HPA axis. In fact, this is not surprising as we have not found relationship between anxiety-like behavior and ACTH and corticosterone response to novel environments in a normal population of rats [50]. Moreover, there is not a clear pattern of differential HPA response to stress among rats genetically selected by the level of anxiety in the EPM [51]–[54]. In contrast to the dissociation between anxiety-like behavior and HPA activity, in the present experiment, the activation of the HPA axis did reflect fear conditioning. Thus, a greater ACTH response to the context previously paired with the cat odor was observed, and this greater response disappeared on the second day concomitantly with the extinction of behavioral conditioning. The enhanced ACTH response was similar in rats only exposed to cat odor as in those also exposed to IMO on day 1, despite the fact that prior IMO exposure per se also increased the ACTH response to the OF. Considering the apparent low level of behavioral conditioning caused by IMO itself, one could argue that this increased ACTH response may be related to long-lasting sensitization of the HPA axis caused by IMO [5], [6] and other severe stressors [55], [56]. However, this is unlikely as sensitization should have been noted in the ACTH response to any novel environment (i.e. EPM) and this was not the case. One alternative explanation is that increased ACTH response to IMO rats reflected some kind of fear conditioning not clearly reflected at behavioral level. If this was the case, it is obvious that no additive effects were found in the animals simultaneously exposed to cat odor and IMO. These results thus open an interesting avenue for further studies on the relationship between the HPA axis and fear conditioning. Corticosterone followed a pattern similar but not strictly identical to ACTH, likely due to the different time-course and the prompt saturation of adrenocortical secretion with intermediate levels of ACTH [1].

Within the framework of the development of putative animal models of post-traumatic stress disorder, the present results did not support the main hypothesis that exposure to a predator odor while immobilized, and therefore without any possibility to escape, can potentiate the negative consequences of exposure to a predator odor. Thus, cat odor-induced fear conditioning and long-lasting unconditioned increase in anxiety were essentially similar regardless of additional exposure to IMO. Nevertheless, considering the complementary effects of each stressor on HPA and emotional sensitization, anxiety and cognitive impairment, the combination of both is of interest. From a more theoretical point of view, exposure to IMO does not appear to interfere with the efficacy of cat odor to induce fear conditioning, suggesting that appropriate attention was paid to the odor even under the exposure to an additional severe stressful situation (IMO). In contrast with this lack of behavioral interaction, activation of the HPA axis during fear conditioning testing suggest that some interference may exist between stressors, a topic that merits to be further explored.
